# *In vivo* Biotinylation Based Method for the Study of Protein-Protein Proximity in Eukaryotic Cells

**DOI:** 10.5195/cajgh.2013.96

**Published:** 2014-01-24

**Authors:** Arman Kulyyassov, Erlan Ramanculov, Vasily Ogryzko

**Affiliations:** 1 National Center for Biotechnology, Almaty, Kazakhstan; 2 Interdisciplinary Instrumentation Center, Nazarbayev University, Astana, Kazakhstan; 3 Institut Gustave Roussy, Villejuif, France

**Keywords:** spaciotemporal structure, protein-protein proximity, spectometry

## Abstract

**Introduction:**

The spatiotemporal order plays an important role in cell functioning and is affected in many pathologies such as cancer and neurodegenerative diseases. One of the ultimate goals of molecular biology is reconstruction of the spatiotemporal structure of a living cell at the molecular level. This task includes determination of proximities between different molecular components in the cell and monitoring their time- and physiological state-dependent changes. In many cases, proximity between macromolecules arises due to their interactions; however, the contribution of dynamic self-organization in generation of spatiotemporal order is emerging as another viable possibility. Specifically, in proteomics, this implies that the detection of protein-protein proximity is a more general task than gaining information about physical interactions between proteins, as it could detail aspects of spatial order *in vivo* that are challenging to reconstitute in binding experiments *in vitro*.

**Methods:**

In this work, we have developed a method of monitoring protein-protein proximity *in vivo*. For this purpose, the BirA was fused to one of the interaction partners, whereas the BAP was modified to make the detection of its biotinylation possible by mass spectrometry.

**Results:**

Using several experimental systems, we showed that the biotinylation is interaction dependent. In addition, we demonstrated that BAP domains with different primary amino acid structures and thus with different molecular weights can be used in the same experiment, providing the possibility of multiplexing. Alternatively to the changes in primary amino acid structure, the stable isotope format can also be used, providing another way to perform multiplexing experiments. Finally, we also demonstrated that our system could help to overcome another limitation of current methodologies to detect protein-protein proximity. For example, one can follow the state of a protein of interest at a defined time after its interaction with another protein has occurred. This application should be particularly useful for studying multistep intracellular processes, where the proximities between proteins and protein properties typically changed in a sequential manner.

**Conclusion:**

This approach has promised in adding temporal dimension in addition to helping reconstruct cell topology in space.

